# Overexpression of LINC01605 promotes early tumorigenic phenotypes in primary human colon epithelial organoids

**DOI:** 10.1016/j.gendis.2026.102125

**Published:** 2026-03-04

**Authors:** Tracy Kongai, Erin E. Johnson, András K. Ponti, Maryssa Shanteau-Jackson, Gail A. West, Tatiana Dombrovski, Christine McDonald, Thomas LaFramboise

**Affiliations:** a Department of Genetics and Genome Sciences, Case Western Reserve University School of Medicine, Cleveland, OH 44106, USA; b Department of Inflammation & Immunity, Lerner Research Institute, Cleveland Clinic, Cleveland, OH 44195, USA; c Department of Biology, John Carroll University, University Heights, Ohio 44118, USA; d Department of Molecular Medicine, Cleveland Clinic Lerner College of Medicine, Case Western Reserve University, Cleveland, OH 44106, USA

Colorectal cancer is the second leading cause of cancer-related death in the United States. Although the molecular pathogenesis of colorectal cancer has been intensively studied, the precise molecular events leading to malignancy remain incompletely understood for most patients. Protein-coding gene mutation drivers and characteristic expression dysregulation patterns have been characterized using large patient cohorts. In contrast, the pathogenic contribution of non-coding genes is less well-studied. We^1^ and others 2, 3, 4, 5 have consistently observed marked up-regulation of LINC01605 in patient tumors and have shown that inhibition of the long non-coding RNA (lncRNA) LINC01605 (also known as lincDUSP) mitigates tumorigenicity in cancer cells, suggesting an oncogenic role for this lncRNA. However, because the analyses relied on knockdown/knockout-based analyses in established cancer cell lines, they left open the question of whether overexpression of LINC01605 in untransformed epithelial cells can contribute to tumorigenic transformation. In the current study, we address this unanswered question by overexpressing LINC01605 in primary human colon epithelial organoids, a model that recapitulates the cellular diversity of the intestinal epithelium far more accurately than traditional cancer cell lines. To our knowledge, no prior work has examined the roles of lncRNAs in colon cancer using an organoid model. Our goal was to determine where LINC01605 up-regulation fits within the canonical genomic alterations summarized in the classical “Vogelgram” model of colon cancer, by assessing whether LINC01605 overexpression induces tumor-related phenotypes that mimic those induced by known driver mutations.

We cloned LINC01605 into an expression plasmid and used it to increase expression levels by transient transfection into organoid cultures (LINC01605-OE) (Fig. 1A). LINC01605 expression levels were confirmed by quantitative reverse transcription PCR and demonstrated ∼20-fold increase over controls (Fig. S1), the level of up-regulation present in colorectal cancer.^1^ We first computed the sizes and numbers of organoids in control and LINC01605-OE cultures at day 3 and day 7 post-transfection. LINC01605-OE organoids formed in significantly greater numbers compared with controls at both day 3 and day 7 (Fig. 1B and C). The increased number of organoids generated from a fixed number of seeded crypts may suggest greater clonogenic potential and self-renewal ability of epithelial stem or progenitor cells. An increase in organoid number is a well-established *in vitro* proxy for enhanced stemness and proliferative capacity, both of which are early hallmarks of tumorigenesis.

**Figure 1:**
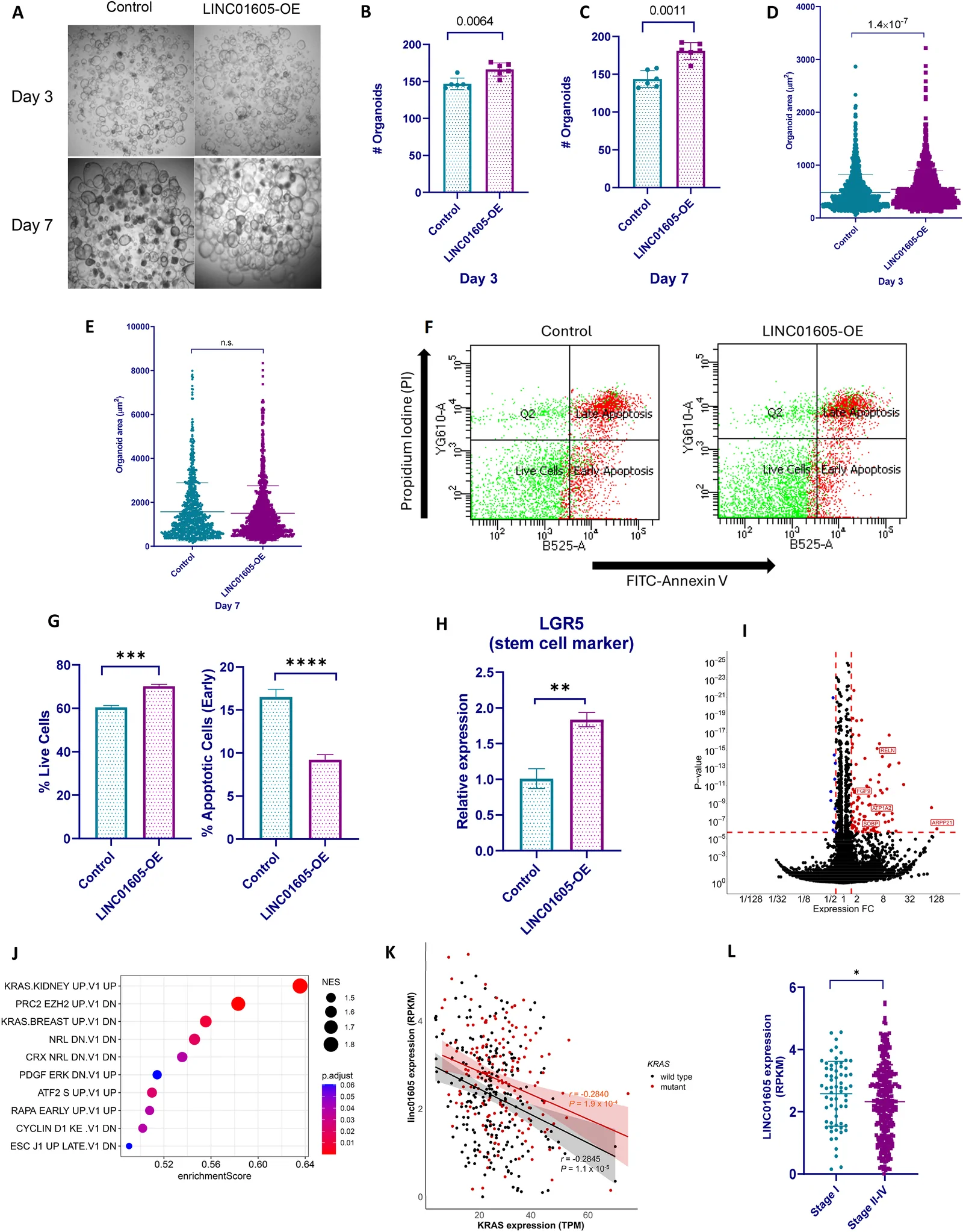
Tumorigenic phenotypes associated with LINC01605 overexpression. **(A)** Representative images of control and LINC01605-OE organoids at day 3 and day 7 post-transfection. **(B**, **C)** Quantification of organoid number per well for each condition and time point (*n* = 6 wells per group). Data presented as mean ± standard deviation. **(D**, **E)** Quantification of organoid size across wells and time points. n.s., not significant. *P* values were calculated using one-sided Wilcoxon tests. **(F**, **G)** Flow cytometry analysis of surface Annexin V in control and LINC01605-OE organoids. ∗∗∗*p* < 0.001 and ∗∗∗*p* < 0.0001. *P* values were computed for differences in proportions of cells using the R function prop.test. **(H)** Quantitative reverse transcription PCR analysis of LGR5 in control and LINC01605-OE organoids. Expression levels were normalized to GAPDH, which served as the internal control, and are presented relative to the control (GFP-transfected). Primer sequences are listed in Table S1. ∗∗*p* < 0.01. **(I)** Volcano plot showing differential gene expression in organoids overexpressing LINC01605. Genes significantly up-regulated (Bonferroni-corrected *p* < 0.05 and fold change > 1.5) and present in the KRAS.KIDNEY_UP.V1_UP gene set are labeled. **(J)** Ten oncogenic gene sets (C6 category) most enriched among genes up-regulated by LINC01605 overexpression. Adjusted *p*-values (p.adjust) were calculated using the Benjamini-Hochberg method. **(K)** Scatter plot showing that KRAS mRNA expression is inversely correlated with LINC01605 expression in colorectal adenocarcinoma samples (*n* = 399 tumors with available expression data for both genes). *P* values were calculated using the R function cor.test. **(L)** LINC01605 mRNA expression across tumor stages in the same cohort. ∗*p* < 0.05. *n* = 373 tumors with available stage and expression information.

Quantitative image analysis also revealed a significantly larger average area in LINC01605-OE organoids compared with controls at day 3 (Fig. 1D), potentially indicating accelerated early growth. By day 7 (Fig. 1E), however, the difference in organoid area between LINC01605-OE and control groups was not statistically significant. The early increase in organoid area observed following LINC01605 overexpression is consistent with its proposed oncogenic role. This apparent transient growth advantage parallels our earlier findings that LINC01605 knockdown in colon cancer cell models reduced growth, viability, proliferation, and colony formation.^1^ Similar results have been observed in other epithelial cancers, where LINC01605 overexpression promotes cell cycle progression, stem-like features, and tumorigenic behavior.^2^^,^^4^ The diminished difference at later timepoints (day 7) suggests that the proliferative effect of LINC01605 may be temporally restricted, potentially constrained by contact inhibition, nutrient limitation, or differentiation-induced growth arrest within dense organoid cultures. Early proliferative bursts followed by plateau phases are characteristic of partially transformed epithelial systems, where oncogenic activation initiates hyperproliferation, but sustained growth and transformation likely require additional cooperating alterations.

Flow cytometric analysis revealed a significant reduction in early apoptotic (Annexin V) cells in the LINC01605-OE group compared with controls (Fig. 1F and G). Correspondingly, the proportion of live cells increased, indicating enhanced cell survival in response to LINC01605 overexpression. The shift toward reduced Annexin V positivity demonstrates that LINC01605 overexpression confers resistance to apoptosis, a hallmark of tumorigenic transformation. These results align with prior evidence that LINC01605 inhibits programmed cell death in epithelial cancers. The decreased apoptotic rate and increased survival observed in LINC0160-OE organoids mirror early oncogenic processes in colorectal tissue.

Further analyses were performed to assess the impact of LINC01605 overexpression on cell cycle progression. Strikingly, the number of cells with fragmented DNA (sub-G0 peak) was lower in the LINC01605-OE group (13.0% *vs*. 34.9%), indicating a higher proportion of viable cells in these organoids (Fig. S2). LINC01605-OE organoids exhibited a notable increase in the G0/G1 phase population, accompanied by modest elevations in S and G2/M phases compared with control organoids. The increased proportion of cells in the G0/G1 phase indicates a potential expansion of a quiescent or progenitor-like cell population. Such cells typically retain proliferative potential while maintaining a less-differentiated state, an attribute commonly associated with intestinal stem and early progenitor compartments. The moderate rise in S and G2/M fractions implies that a subset of cells remains actively cycling, suggesting that LINC01605 supports both cell cycle readiness and progression.

We next quantified expression levels of the stem cell marker leucine-rich repeat-containing G-protein-coupled receptor 5 (LRG5), goblet cell marker mucin 2 (MUC2), and enterocyte marker sucrase-isomaltase (SI). Compared with control normal organoids, LINC01605-overexpressing organoids showed increased expression of LGR5 (Fig. 1H), with a trend towards decreased MUC2 (Fig. S3A) and no change in SI (Fig. S3B). These transcriptional changes may indicate that LINC01605 promotes a stem-enriched, less-differentiated organoid phenotype. However, the trends are mostly suggestive, given the modest changes in MUC2 and SI.

To assess transcriptional impact of LINC01605 overexpression, we performed RNA sequencing of control and LINC01605-OE organoids (Fig. 1I). Genes up-regulated upon LINC01605 overexpression were most significantly enriched in the gene set KRAS.KIDNEY_UP.V1_UP, which contains genes up-regulated in kidney cancer when oncogenic KRAS is expressed (Fig. 1J; Fig. S4; up-regulated genes in this set are indicated in Fig. 1I). This enrichment suggests that high LINC01605 expression results in a transcriptional profile similar to that induced by oncogenic KRAS. We hypothesized that, if LINC01605 overexpression mimics the effects of oncogenic KRAS, then their expression levels should be inversely correlated. Specifically, LINC01605-driven over-proliferation would be most pronounced in cells with low oncogenic KRAS expression. To investigate, we analyzed KRAS and LINC01605 mRNA expression data from the colon adenocarcinoma cohort of the Cancer Genome Atlas (TCGA). Across the cohort, we observed a strong negative correlation between expression of the two genes (Spearman *ρ* = −0.26, *p* = 1.1 × 10^−5^; Fig. 1K), which was even stronger in tumors harboring mutant KRAS (Spearman *ρ* = −0.28, *p* = 1.9 × 10^−4^; Fig. 1K).

The data presented above support the hypothesis that LINC01605 up-regulation contributes to the early stages of tumor initiation. To determine whether this pattern is reflected in patient samples, we analyzed LINC01605 levels across colon adenocarcinoma stages using TCGA data. We stratified tumors by clinical stage, reasoning that molecular alterations functioning early in tumorigenesis should be more prominent in stage I, whereas later stage-associated alterations should increase in stages II-IV. Indeed, LINC01605 expression is significantly higher in stage I tumors compared with stages II-IV (Fig. 1L). This observation, though strictly correlative, is consistent with LINC01605 up-regulation occurring early during tumorigenesis.

In conclusion, LINC01605 overexpression reprograms normal human colonic organoids toward a proliferative, stem-enriched, and apoptosis-resistant state, capturing key molecular hallmarks of early colorectal cancer. This work provides a foundation for understanding the contribution of noncoding RNAs to epithelial transformation. It reinforces the value of organoid systems as translational tools for dissecting cancer initiation.

## CRediT authorship contribution statement

**Tracy Kongai:** Writing – review & editing, Writing – original draft, Visualization, Validation, Methodology, Investigation, Formal analysis, Data curation, Conceptualization. **Erin E. Johnson:** Writing – review & editing, Validation, Resources, Investigation. **András K. Ponti:** Writing – review & editing, Resources, Methodology, Investigation, Formal analysis. **Maryssa Shanteau-Jackson:** Resources, Methodology, Investigation, Formal analysis. **Gail A. West:** Resources, Methodology. **Tatiana Dombrovski:** Writing – review & editing, Software, Formal analysis. **Christine McDonald:** Writing – review & editing, Writing – original draft, Supervision, Resources, Project administration, Methodology, Investigation, Conceptualization. **Thomas LaFramboise:** Writing – review & editing, Writing – original draft, Supervision, Project administration, Funding acquisition, Conceptualization.

## Funding

This work was supported by the 10.13039/100000002US National Institutes of Health grants R01LM013067, R01CA217992, R21CA249138 (to T.L.), and philanthropic funds from the McDonald Family Trust (to C.M.).

## Conflict of interests

T. LaFramboise is an Editorial Board member for *Genes & Diseases* and was not involved in the editorial review or the decision to publish this article. C. McDonald serves as a scientific advisor for PALA Pharmaceuticals, Inc, which was not involved in the study design, conduct, data analysis, or reporting of the research presented in this report. All other authors have no conflict of interests to declare.
