# Supplementary material for: Overexpression of LINC01605 promotes early tumorigenic phenotypes in primary human colon epithelial organoids
Source: Genes Dis. 2026 Mar 4;14(3):102125. doi: 10.1016/j.gendis.2026.102125 (PMC13734857; doi:10.1016/j.gendis.2026.102125)
Supplement: Supplementary file 1 [file mmc1.docx]

**Supplemental information**

**Materials and Methods**

**LINC01605 isoform analysis**

Colon cancer cell lines V703, HCT116, and HT29 were used to determine the predominant isoform of LINC01605 expressed in CRC. V703 cells were maintained in MEM2+ media supplemented with 2% FBS, 2 mM glutamine, 1 μg/mL hydrocortisone, 10 μg/mL insulin, 0.86 ng/mL selenium, 2 μg/mL transferrin, and 50 μg/mL gentamicin. HCT116 and HT29 cells were cultured in Dulbecco’s Modified Eagle’s Medium (DMEM) supplemented with 10% FBS, 100 U/mL penicillin, and 100 μg/mL streptomycin. All cell lines were maintained at 37°C, 5% CO₂, with media changes every other day. Cells were passaged approximately every 5 days using trypsinization. Isoform-specific primers for each LINC01605 transcript were designed from NCBI (https://www.ncbi.nlm.nih.gov/) reference sequences and verified by NCBI BLAST and Primer3Plus (Table S2). PCR was performed with Phusion High-Fidelity DNA Polymerase (New England Biolabs), and products were run on a 1% agarose gel. GAPDH served as an internal control. The predominant isoform in each cell line was identified by its presence or absence, and the isoform detected in all cell lines was selected for subsequent cloning and functional assays.

**Generation of LINC01605 expression vector**

The pcDNA3.1+ vector was obtained from Invitrogen (cat# V79020). The LINC01605 transcript variant 4 (NR_170187.1) gene Block fragment (gBlock) was ordered from Integrated DNA Technologies (IDT). The plasmid was constructed by assembling overlapping fragment ends using Gibson assembly according to the manufacturer’s protocol (New England Biolabs Gibson Assembly Master Mix; cat# E2611S). Products were subsequently sequenced using the T7 promoter sequencing primer. LINC01605 primers were designed using Primer3plus. Sequences are provided in Table S1.

**Human colon organoid culture**

Primary human colon organoids were established from epithelial crypts isolated from de-identified, tumor-adjacent (>6 cm from tumor site) normal tissue collected under Cleveland Clinic Institutional Review Board approved protocols (IRB# 17-1167 and IRB# 12-383) from a 62-year-old female patient undergoing resection of colon adenocarcinoma. Intestinal crypts were isolated as previously described (1) and organoid culture followed previously established protocols (2, 3), with minor modifications to optimize yield and viability.

**Organoid transfection**

Transient transfection of human colon organoids was performed using the P3 Primary Cell 4D-Nucleofector™ X Kit S (Lonza cat# V4XP-3032) following the manufacturer’s protocol, optimized for epithelial organoids. The organoids were first mechanically disrupted in PBS–EDTA buffer to release single cells and small clusters, followed by three washes in cold PBS to remove Matrigel residue. The resulting organoid pellet was resuspended in P3 Primary Cell Nucleofector Solution supplemented with Supplement 1, ensuring a homogeneous cell suspension. The cell–DNA mixture was transferred into cuvettes for 4D-Nucleofector™ electroporation using a program optimized for human intestinal epithelial cells. After nucleofection, organoids were immediately resuspended in the recovery medium and plated in Matrigel domes to facilitate re-aggregation and growth. Transfection efficiency and cell viability were assessed after 48 hours. Successful LINC01605 overexpression was confirmed by RT-qPCR analysis using LINC01605 transcript variant 4 primers.

**Organoid quantification**

To assess growth and density, LINC01605-overexpressed and control organoids were plated in a 24-well plate. Images of organoids suspended in Matrigel domes were acquired using an EVOS FL microscope with a 2X bright-field objective. For organoid quantification, captured images were analyzed using ImageJ software. The software was used to measure organoid size (area in microns) and to count the number of organoids present in each Matrigel dome. Organoids smaller than 100 µm^2^ were omitted from size and count statistics.

**Flow cytometry experiments**

The organoids in Matrigel were mechanically disrupted in a cold PBS-EDTA buffer, washed three times, and then washed in cold 1X PBS until no Matrigel residual was visible, leaving only the organoid pellet. For the apoptosis analysis, the organoids were processed using the FITC Annexin V Apoptosis Detection Kit following the manufacturer’s instructions (ThermoFisher cat# V13242) and analyzed on a flow cytometer using the B525 channel (FITC-Annexin V) and the YG610 channel (Propidium Iodide, PI). Differences in proportions of apoptotic and live cells between the control and LINC01605-overexpressed samples were statistically tested using the prop.test function in R (version 4.5.0).

For cell cycle analysis, PI staining was used to quantify the proportion of cells in the G0/G1, S, and G2/M phases. Organoids were harvested mechanically as described above and fixed with methanol. Following fixation, cells were treated with RNase A to eliminate RNA and subsequently stained with PI. The stained cells were then analyzed using a Flow Cytometry Discover A8, with DNA content measured based on fluorescence intensity. This analysis enabled the discrimination and quantification of cells in different phases of the cell cycle (G0/G1, S, and G2/M) according to their DNA content.

**RNA isolation, cDNA preparation, and qRT-PCR analysis**

For qRT-PCR experiments, organoids were resuspended in Corning cell recovery solution (Corning cat# 354253) to dissociate the Matrigel and obtain the cell pellets. RNA extraction was performed from the cell pellet according to the manufacturer's instructions using the Qiagen RNeasy Mini Kit (cat# 74106). RNA quality was assessed using a NanoDrop spectrophotometer. Complementary DNA (cDNA) synthesis was performed using the Bio-Rad iScript cDNA synthesis kit (cat# 1708891). qRT-PCR was performed in triplicate using iTaq universal SYBR Green supermix (cat# 1725124) on a Bio-Rad CFX96 Real-Time system. Data were analyzed using the comparative cycle threshold (C_q_) method to obtain relative expression quantities after normalization to GAPDH mRNA levels. Primer sequences for LGR5, MUC2, and SI were previously published (1, 4, 5) and are provided in Table S1.

**RNAseq**

RNA extraction was performed as described above in triplicate from both control and LINC01605 overexpressing organoids. The extracted RNA was sequenced at the Genomics Core at Case Western Reserve University. Raw paired fastq files from the RNA sequencing were first quality-checked and of trimmed low-quality bases and adapter sequences using Trim Galore (v0.6.10) with default settings. The trimmed reads were aligned to the human reference genome (GRCh38/hg38) using the STAR aligner (version 2.7.9a) with default parameters for paired-end data. The aligned reads were then sorted by genomic coordinates and indexed using SAMtools (v1.19.2).

Gene quantification was performed using FeatureCounts from the Subread package (v2.0.3), using GTF annotations from Ensembl. Differential gene expression analysis was performed on the count matrix with the DESeq2 package (v1.38.3).

For gene set enrichment analysis (GSEA), a ranked gene list was created based on log2 fold changes. Enrichment analysis was performed using the clusterProfiler R package (v4.6.2), with the C6 (Oncogenic Signatures) gene sets from the Molecular Signatures Database (MSigDB), accessed through the msigdbr R package (v10.0.1). Significantly enriched gene sets were identified using a Benjamini-Hochberg adjusted p-value threshold of 0.05.

**Public data**

Colon adenocarcinoma (COAD) gene expression data from the Cancer Genome Atlas (TCGA) were downloaded from cBioPortal (cbioportal.org (6)) and Genomic Data Commons Data Portal (https://portal.gdc.cancer.gov/).

**Statistical analysis**

Data are presented as mean ±SD. Experiments were performed in triplicate unless otherwise specified. Statistical significance between two groups was assessed using one-sided Wilcoxon tests, except for comparing cell count proportions in the flow cytometry experiments, where the R functions prop.test and cor.test were used. The tests were one-sided since our hypothesis *a priori* was that LINC01605-OE organoids would have higher numbers, larger areas, more live cells, lower apoptosis, etc. That is, the alternative hypothesis in the statistical tests is one-sided. A p-value of < 0.05 was considered statistically significant for individual tests.

**References**

1.	Sato T, Stange DE, Ferrante M, Vries RG, Van Es JH, Van den Brink S, et al. Long-term expansion of epithelial organoids from human colon, adenoma, adenocarcinoma, and Barrett's epithelium. Gastroenterology. 2011;141(5):1762-72.

2.	Miyoshi H, Stappenbeck TS. In vitro expansion and genetic modification of gastrointestinal stem cells in spheroid culture. Nature protocols. 2013;8(12):2471-82.

3.	Pleguezuelos-Manzano C, Puschhof J, van den Brink S, Geurts V, Beumer J, Clevers H. Establishment and Culture of Human Intestinal Organoids Derived from Adult Stem Cells. Curr Protoc Immunol. 2020;130(1):e106.

4.	Co JY, Margalef-Catala M, Li X, Mah AT, Kuo CJ, Monack DM, et al. Controlling Epithelial Polarity: A Human Enteroid Model for Host-Pathogen Interactions. Cell reports. 2019;26(9):2509-20 e4.

5.	Drost J, van Jaarsveld RH, Ponsioen B, Zimberlin C, van Boxtel R, Buijs A, et al. Sequential cancer mutations in cultured human intestinal stem cells. Nature. 2015;521(7550):43-7.

6.	Cerami E, Gao J, Dogrusoz U, Gross BE, Sumer SO, Aksoy BA, et al. The cBio cancer genomics portal: an open platform for exploring multidimensional cancer genomics data. Cancer discovery. 2012;2(5):401-4.

**Supplementary Figures**

**Fig. S1.** RT-qPCR analysis visualizing the level of LINC01605 overexpression after transfection of LINC01605; primer sequences are provided in Table S2. LINC01605 levels are normalized to GAPDH expression and graphed as expression relative to control cultures.

**Fig. S2.** LINC01605-OE organoids exhibit a notable increase in the G0/G1 phase population, accompanied by modest elevations in S and G2/M phases, compared with control organoids. Cells with fragmented DNA (sub-G0 peak) are indicated with red arrows.

**B**

**A**

**Fig. S3.** RT-qPCR analysis of **(A)** MUC2, and **(B)** SI expression in control and LINC01605 overexpressing organoids. Expression levels were normalized to GAPDH, which served as the internal control, and are presented relative to the control (GFP-transfected). Primer sequences are listed in Table S2.

**Fig. S4.** Enrichment plot for KRAS.KIDNEY_UP.V1_UP pathway. FC, fold change; NES, normalized enrichment score.
